# Supplementary material for: Learning curve and surgical outcome of robotic assisted colorectal surgery with ERAS program
Source: Sci Rep. 2022 Nov 29;12:20566. doi: 10.1038/s41598-022-24665-w (PMC9709162; doi:10.1038/s41598-022-24665-w)
Supplement: Supplementary file 1 — Supplementary Information 1. [file 41598_2022_24665_MOESM1_ESM.zip › LSC Raw data-Ñ[▒K/069/N_ñw╝╨░O▒Kñσ.pdf]

W95 / W95

臺中榮民總醫院

[redacted]

基本資料

- 室別： 二醫手術室-31室，手術科別： CRS，手術類別： 常規，日期： 2020/02/19
- 進入時間： 0745 ； 麻醉開始時間： 0800 ； Time Out時間： 0905 ； 劃刀時間： 0906 ； 手術結束時間： 1310 送出時間： 1315 ；
- 麻醉方式： GE
  診斷： S colon ca
  手術名稱： Laparoscopic Anterior resection and anastomosis - malignant
  術前預防性抗生素：
  - 抗生素名稱 ： Veterin 1GM IVD/由麻醉護理師給予
  - 時間： 0850
  - 上次簽章: 朱詠暄

  , 追加預防性抗生素：
  - 抗生素名稱一： Veterin 1GM IVD/由麻醉護理師給予
  - 時間： 1250
  - 記錄者： 朱詠暄
- 手術醫師： 林俊余
- 麻醉醫師： VS 陳永銘
-
  - 刷手護士A ： 彭馨慧，時間： 0745-1250，刷手護士B ： 戴宛榛，時間： 0745-1250
  - 流動護士A ： 朱詠暄，時間： 0745-1250

皮膚狀況

A-V Shunt： 無
術前： 完整
記錄者： 上次簽章: 朱詠暄
術後： 完整
記錄者： 朱詠暄

手術準備

手術臥式 單一手術臥式

-
  - 手術臥式： 截石臥式
  - 使用附件： 溫毯 38 ℃，頭圈，腳架
  - 皮膚準備： 10%Aq Betadine，Hibiscrub，其它： 2 %chlorhexidine
  - 電燒： 使用電燒: 電極片位置： 其它： 電燒墊，記錄者： 上次簽章: 朱詠暄

止血帶T： 無

植入物

無

標本送檢

- 細菌培養 無
- 細胞學 無
- 冰凍切片 無
- 病理標本
  組織名稱一： S-colon
  ,
  組織名稱二： distal cutend
  ,
  組織名稱三： IMA root
- 截肢 無

標本交付 標本： ? 袋，標本： 0 盒，標本： 0 瓶，細胞學： 0 管，Culture Tube： 0 管，被交付醫師： CR 黃俋

霖，

- 記錄者： 朱詠暄
- 時間： 1315

傷口術後敷料

傷口敷料：

- 請於圖上標示部位：

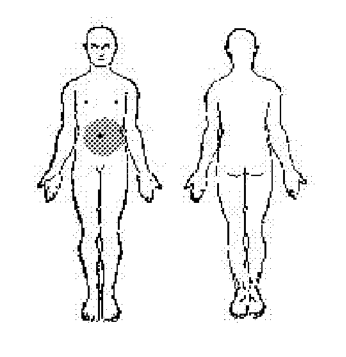

腹部六切口

- 傷口敷料： mepore

導管

導管	天數	導管資訊	插入日期	插入時間	更換日期	拔除日期	拔除時間	拔除原因	預計拔除/更換	預計拔除/更換日期
Foley(1)	1	號碼:16 Fr.; 部位:膀胱; 註記:水球10cc;	2020/02/19	0840						
A-line (1)	1	號碼:20號 ; 部位:左手;	2020/02/19	0845						
Sump NG Tube(1)	1	號碼:16 Fr.; 部位:胃;	2020/02/19	0850						

用物計數

- 手術中出血量： minimal c.c.
- 手術中尿量： 1300 c.c.
-
    - 其它： 無

用物計數： 多切口同時縫合

- 1.器械： 正確，2.縫針： 正確，3.刀片： 正確，4.紗布： 正確，5.腹部墊： 未使用，6.紗球： 未使用，7.PEANUTS ： 未使用，8.紗條： 未使用，9.絲帶： 未使用，10.針頭： 未使用，11.Vessel loop： 未使用，12.Boots： 未使用，13.Jelco： 未使用，14.棉條： 未使用，
    - 第一次：
        - 刷手護士 ： 彭馨慧
        - 流動護士： 朱詠暄
        - 時間： 1230

      ，
    - 第二次：
        - 刷手護士 ： 彭馨慧
        - 流動護士： 朱詠暄
        - 時間： 1250

      ，
    - 第三次：
        - 刷手護士 ： 彭馨慧
        - 流動護士： 朱詠暄
        - 時間： 1300

備註

備註： NIL

- 記錄者： 朱詠暄 上次簽章: 朱詠暄
- 時間： 1315

填寫護理人員 朱詠暄

正式摘要

W95 / W95

doctorRank:B
